# Psychosocial and Sexual Implications of Repaired Cleft Lip in an Adult Male

**DOI:** 10.7759/cureus.8563

**Published:** 2020-06-11

**Authors:** Jason Gandhi, Omar Seyam, Raymond Liang, Noel L Smith, Sardar A Khan

**Affiliations:** 1 Department of Physiology and Biophysics, Renaissance School of Medicine at Stony Brook University, Stony Brook, USA; 2 Medical Student Research Institute, St. George's University School of Medicine, St. George's, GRD; 3 Primary Care, Foley Plaza Medical, New York, USA; 4 Department of Urology, Renaissance School of Medicine at Stony Brook University, Stony Brook, USA

**Keywords:** psychosocial, sexual dysfunction, lips, cleft lip, cleft palate, speech disorders, psychological adjustment

## Abstract

We describe the case of a 32-year-old man with repaired cleft lip complaining of sexual difficulties, which were determined to be multifactorial in origin. A negative body impression, anatomical difficulty in engaging in sexual acts, the indirect impact of secondary complications of cleft lip, and the negative perception of his dysmorphia from potential sexual partners led to his current state. Cleft lip and palate (CLP) are the most common malformations of the craniofacial region. The malformations of anatomical structures involved in CLP can manifest through several variations of clinical features and phenotypes, typically affecting hearing, social integration, speech, and feeding. From birth to the end of growth, a multidisciplinary approach involving orthodontists, speech therapists, psychologists, and social workers is essential for adequate management, even after surgical repair. This case report illustrates the critical importance of the functionality of normal lips essential for sexual function and psychosocial issues encountered in a patient with repaired cleft lip, as this issue likely has a higher prevalence than the literature actually suggests. Adding a psychiatrist on the panel for pediatric reconstructive surgery teams and post-repair rehabilitation would be essential to managing potential sexual and psychological issues into adulthood.

## Introduction

Cleft lip and palate (CLP), or orofacial cleft, are birth defects where certain facial tissues do not join during development. If mismanaged, CLP can lead to poor quality of life from childhood to adulthood. Aside from feeding and speech problems, other overlooked challenges are possible. Such challenges may include a high anxiety disorder rate in adolescent patients and a decrease in the quality of their family life [1,2]. Conversely, in a cross-sectional study, adults with a cleft had a significantly higher quality of life when compared to adults without a cleft, possibly due to their resiliency [3]. Herein we describe a patient who faced sexual handicap secondary to cleft lip and its related psychosocial and anatomical issues.

## Case presentation

A 32-year-old male consulted primary care for sexual difficulties. He had a history of a unilateral left-sided incomplete cleft lip without cleft palate that was repaired several times by a surgeon. His clinical history was a deformity of upper lip which interfered with sexual foreplay and other sexual activity. He was self-conscious about his facial appearance and highly recognizable dysfunction of his lips during sexual activity. He claimed to have difficulty dating and getting married because of his conspicuous lip deformity. The patient was a non-smoker, occasionally drank alcohol, and did not chew tobacco. Taste and hearing were normal. He had difficult articulation of speech. He was unable to whistle and had difficulty in spitting and pursing his lips. The patient also had intermittent episodes of difficulty keeping food and fluids in his mouth. He believed these issues barred him from attaining and keeping a sexual partner.

During physical examination, the patient was found to be well nourished. Examination of his upper lip demonstrated three healed scars, two on the right and one on the left, as well as a shortened and wrinkled lip. The wounds were well-healed without sinuses or keloids. Movement of the upper lip was restricted with decreased sensation to touch and temperature. The color of the lip was unremarkable. The philtrum was irregular, and the vermilion border was upturned. Attempts to seal his lips together were restricted. The Cupid's bow was asymmetrical. The right and left nasolabial folds were present but irregular. The frenulum of the upper lip was scarred and the lip was ankylosed. The right commissure was irregular and drooping; attempts to close the mouth by approximating both of the lips resulted in a snarl. Examination of the lower lip revealed that innervation, sensation, and movement were within normal limits. The appearance of the lip was unremarkable. The buccal frenulum was normal appearing. The mentolabial sulcus of the chin was unremarkable. Upon examination of the oral cavity, his teeth were irregularly placed. The gingiva was normal. Movement and sensation of the tongue were unremarkable. The uvula, hard and soft palate, as well as palatine tonsils were unremarkable. Complete blood count, basal metabolic panel, free and total testosterone, prolactin, and sex hormone binding globulin were within normal limits.

The patient was referred to a team of plastic surgeons for corrective options for his deformity as well as a psychologist for improving self-image.

## Discussion

To understand how CLP can handicap sexual activity, we acknowledge some other conditions that physically challenge the act of sex in Table 1. Subsequently, Table 2 lists congenital and acquired lip conditions that indirectly challenge the act of sex, specifically the erotic stimulation preceding sexual intercourse [4]. Knowing the full extent of these conditions can help us understand how secondary complications of CLP can further potentiate sexual difficulties. CLP patients may borrow insight from similarly afflicted patients to learn a new strategy for coping with their sexual challenges. The movement and innervation of the lips are indispensable for autonomic arousal prior to sexual activity, as well as speech, hearing, and feeding [5]. Loss of lip movement, ankyloses, deformity, scarring, and denervation results in difficulty in foreplay related to sexual activity. In addition, the psychosexual impact in terms of subjective embarrassment and shame for their condition can diminish arousal, particularly in the presence of a sexual partner.

**Table 1:** Physically significant conditions that can interfere with sexual activity.

Condition
Lip disorders
Trauma
Iatrogenic injuries (e.g., surgical sites)
Neoplasms
Dental disorders
Sleep apnea
Allergies
Radiation injury
Body piercing

**Table 2:** Lip conditions functionally or cosmetically interfering with sexual activity.

Condition
Ankyloses
Keloid
Lip prints, lip clefts, lip pits, Fordyce granules
Venous lakes
Caliber persistent artery
Morsicatio labiorum
Mucoceles
Angular cheilitis
Herpes labialis
Gingivostomatitis
Verrucous papules
Molluscum contagiosum
Syphilis
Exfoliative cheilitis
Atopic cheilitis
Allergic contact cheilitis
Discoid lupus erythematosus
Sjogren disease and xerostomia
Cheilitis glandularis
Psoriasis
Drug-associated cheilitis
Actinic cheilitis
Squamous cell carcinoma of the lip

Adolescents who have a CLP are at a higher risk for psychosocial problems, which include peer relationships, self-concept, and appearance [6-9]. Particularly in girls, self-concept may be affected negatively with the presence of CLP. However, it was found that children from the ages of three to five years were found to have a self-concept that is similar to the others that do not have a cleft. There have been higher levels of anxiety and more dissatisfaction among children through relationships with their peers as they grow older which can be due to possible speech impediments and the associated stigma of visible deformities [10]. During preschool years, the relationship between a child’s self-concept and parental attitudes is very important. Consequently, it was found that there were reduced social skills in children whose mothers had high levels of stress [11]. The development of social skills begins to be impacted by their peers and parental support when reaching the time early elementary years. When the children reach the ages of 13 through 19 years, the peer groups become a major focus of attention. Appearance is one of the important characteristics to adolescents above humor and intelligence [12].

In order to repair the CLP defect, surgery is first line in improving conditions based on a child’s circumstance. There may be follow-up surgeries to improve the appearance of the nose and lip as well as speech even after the initial cleft repair. There are several procedures, which include cleft lip repair, cleft palate repair, ear tube surgery, and surgery, to reconstruct appearance [13]. Though initial reconstructive surgeries may fix functional impairment to an extent, psychosocial and sexual impairment into adulthood may elicit the need for further cosmetic repair by plastic surgeons, as with our patient. A cross-sectional study objectively measuring scarring and three-dimensional facial asymmetry after surgical correction of unilateral cleft lip and unilateral cleft palate in children found that visibility of the scar was significantly correlated with lower self-esteem and higher trait anxiety [14].

The treatments for CLP are meant to improve the speech, as well as the ability to eat and to hear. Post-surgical scars of cleft lip patients can constrain lip activity, stunting maxillary growth. Our patient may have benefited from fractional CO_2_ laser therapy to preserve lip activity and reduce electromyographic activity of the upper lip at rest [15]. A team of physicians and experts, utilizing a coordinated approach as noted in Table 3, is essential to care for children who have CLP [16]. Longitudinal studies have looked at psychological adjustment to CLP to determine clinical utility [17]. In addition, prenatal diagnosis facilitates timely counseling of the parents by the CLP team and increases preparedness for the family which improves the child’s quality of treatment enabling a quality and standard of life as close to the norm as possible [18]. Further studies conceptualizing and measuring psychosocial adjustment to CLP are warranted with a particular focus on developmental trajectory, behavior, emotional well-being, social experiences, satisfaction with appearance and treatment, and sexual implications into adulthood [19]. There must be increased efforts to obtain data from large, representative, and longitudinal samples, which are comparable across studies and inclusive of the patient perspective [15].

**Table 3:** Coordinated treatment strategy for cleft lip and palate.

Personnel	Description
Feeding specialist	Assesses and manages feeding issues related to a cleft diagnosis [16]
Nurse coordinator	Coordinates the multispecialty care and management of the patient [19]
Plastic and oral surgeon	Executes surgical procedures related to the cleft lip/palate, orthognathics, velopharyngeal insufficiency, and nose [16]
Otolaryngologist	Assesses auditory issues, tympanic membrane management [16]
Dentist	Prevents and treats tooth and gum disorders and diseases [16]
Orthodontist	Corrects irregularities of tooth position [16]
Prosthodontist	Replaces teeth and makes dental and alveolar molding devices [16]
Geneticist	Assesses and diagnoses genetically linked diseases and disorders [16]
Speech therapist	Diagnoses and treats disorders of speech [20]
Social worker	Provides social services such as insurance needs [16]
Psychologist	Limit social stigma, self-image, and social acceptance concerns [19]

## Conclusions

Notably, children who are affected with CLP and require multidisciplinary care until adulthood possess a higher level of mortality and morbidity. There is a burden imposed upon the individual which affects them psychosocially, although rehabilitation can be achieved through quality care. Children who are born with these defects are cared for by nursing, speech therapy, orthodontics, and plastic surgery. Children born with CLP must have a multifaceted treatment approach into adulthood so that psychosocial complications, such as language development, sexual handicap, and relationship difficulty, do not develop in the future. Although surgery is an option in adulthood, these issues must be addressed early on to prevent lasting psychosocial problems. For these problems, support groups can be a source of guidance from others who have experienced or are experiencing a similar plight.
